# Cognitively and physically demanding exergaming to improve executive functions of children with attention deficit hyperactivity disorder: a randomised clinical trial

**DOI:** 10.1186/s12887-016-0757-9

**Published:** 2017-01-10

**Authors:** Valentin Benzing, Mirko Schmidt

**Affiliations:** Institute of Sport Science, University of Bern, Bremgartenstrasse 145, 3012 Bern, Switzerland

**Keywords:** ADHD, Active video gaming, Physical exercise

## Abstract

**Background:**

Attention deficit hyperactivity disorder (ADHD) is one of the most common mental disorders observed in childhood and adolescence. Its key symptoms — reduced attention, poor control of impulses as well as increased motor activity — are associated with decreased executive functions performance, finally affecting academic achievement. Although drug treatments usually show some effect, alternative treatments are continually being sought, due to lack of commitment and possible side effects. Cognitive trainings are frequently used with the objectives of increasing executive function performance. However, since transfer effects are limited and novelty and diversity are frequently ignored, interventions combining physical and cognitive demands targeting a broader range of cognitive processes are demanded.

**Methods:**

The aim of the study is to examine the effects of a cognitively and physically demanding exergame on executive functions of children with ADHD. In a randomised clinical trial, 66 girls and boys diagnosed with ADHD (age 8–12) will be assigned either to an 8-week exergame intervention group (three training sessions per week à 30 min) or a waiting-list control group. Before and afterwards, the executive function performance (computer-based tests), the sport motor performance and ADHD symptoms will be assessed.

**Discussion:**

The current study will offer insights into the effectiveness of a combination of cognitive and physical training using exergaming. Positive effects on the executive functions, sport motor performance and ADHD symptoms are hypothesized. Beneficial effects would mean a large degree of scalability (simple and cost-effective) and high utility for patients with ADHD.

**Trial registration:**

KEK BE 393/15 (March 8, 2016); DRKS00010171 (March 14, 2016)

## Background

Children and adolescents with attention deficit hyperactivity disorder (ADHD) display an increased risk of suffering from long-term academic, work-related and social impairments, which are linked to the key symptoms of ADHD: impaired attention, hyperactivity and lack of control of impulses [1]. ADHD is one of the most common disorders in childhood and adolescence with an estimated prevalence of 3–7% [2, 3], affecting more boys than girls [4]. The key symptoms usually already occur at pre-school age and may persist into adulthood [5]. Neurocognitive consequences of the disorder may take the form of deficient executive functions and motor deficiencies [6–8].

Although a large proportion of the symptoms can be reduced by medication, the main method of treatment [9], medications are also associated with a series of difficulties. Not all patients respond to treatment, about one third does not respond or responds poorly to treatment [10–12]. In some cases, side effects [13], low compliance [14] and as yet largely unknown long-term sequelae [15] occur. For these reasons, alternative methods of treatment are constantly being sought, which can improve the main functional deficits associated with the symptoms of ADHD. The prevailing explanation for the development of the key symptoms of ADHD is seen in a primary deficit in the executive functions [16].

Pronounced ADHD symptoms (attention deficit, hyperactivity, impulsiveness) are negatively correlated with the executive function performance [17–22]. Executive functions are viewed as meta-cognitions which are necessary for the top-down modulation of fundamental cognitive processes and therefore for carrying out goal-oriented activities [23]. Three fundamental processes can be seen as forming the core of the executive functions: *inhibition*, *working memory* and *cognitive flexibility* [24]. The executive functions are involved in all patterns of goal-oriented thought and behaviour and therefore control all behaviour that is relevant to learning [23], which explains the strong connection between learning success and executive functions in children in general [25] as well as in children with ADHD [26]. Promoting executive functions could therefore be a key pillar in a successful treatment strategy aiming to reduce the key symptoms of ADHD.

The positive influence of increased physical exercise on a wide range of areas of cognition has been repeatedly demonstrated for all age groups [27, 28]. In particular, inhibition and cognitive flexibility seem to profit from it [29]. Since children with ADHD often display deficient motor skills and motor control in addition to a functional deficit in their executive functions [30], increased physical exercise might be particularly important to this study population. Promising effects of physical activity, mostly based on acute studies, have been demonstrated in children with ADHD [31], and these might be explained by a potentially strong link between sensory motor skills, higher order cognitive processes and academic achievement [32]. Aside from these cross-sectional studies, experimental studies aiming to examine the longer-term influence of physical exercise on cognitive performance in childhood are rare [33] and even more so for children with ADHD [34].

The few existing intervention studies mostly reveal positive effects on cognitive performance [35–41], whereby multimodal treatment methods in particular are attributed with a greater efficacy and broader transfer effects (also to health and well-being). In this context, qualitative characteristics of the physical activity, such as adaptiveness to the individual performance level and cognitive demands, are attributed with playing a central role [42]. The fact that a training programme needs to be individually customised and adaptive in order to have positive effects on the executive functions is generally accepted in the field of cognitive training [43, 44].

The effectiveness of cognitive training programmes has increasingly been studied in children with ADHD in recent years [45]. This was mostly done using computerised, adaptive training programmes, aiming at attention processes, working memory or inhibition, for example. Neuropsychological deficits are to be reduced and transfer effects to the symptoms of ADHD and the general level of functioning are to be facilitated [46]. However, such effects seem to be limited to improvements in working memory performance [45]. Since most training programmes only aim at one area of the executive functions (e.g. working memory) [47], the transfer effects to other fundamental processes and to the ADHD symptoms as a whole continue to be a matter of controversy [48]. Thus, for example, improved working memory only leads to an improved learning performance if the child is able to block out (inhibit) irrelevant information. Cortese et al. [45] therefore call for innovative treatment programmes that increase transfer effects and that target multiple neuropsychological processes.

Just as there have been promising developments towards making cognitive training programmes as multifaceted as possible, the idea that highly varied, cognitively demanding sports activities offer an additional benefit for the executive functions [49] is increasingly being pursued in the field of physical training programmes too [23, 50]. A recent study [51] was able to demonstrate that a 6-week, cognitively demanding sports game intervention, but not a pure aerobic exercise intervention, had a positive influence on the cognitive flexibility of primary school children. In order to have the largest possible effect, Moreau and Conway [52] recommend a combined physical and cognitive training programme. This should ideally be carried out in the form of an ecologically valid but nevertheless controllable study.

An innovative combination of physical and cognitive training that takes into account the recommendations of Moreau and Conway [52] can be achieved with the help of “exergaming”. Exergaming is a portmanteau of “exercise” and “gaming” and refers to a new genre of interactive video games, which allow the gaming experience to be extended to the entire body [53]. In the light of existing motivational problems and a lower level of positive reinforcement, children with ADHD often find conventional training programmes boring and tiring [47]. “Gamification”, on the other hand, i.e. the use of game elements in non-game contexts [54, 55], appears to have a positive effect on motivation and training outcomes [56, 57]. This connection leads to a hybrid form that lies between a physical and cognitive training programme and a video game. This form of training ensures adaptivity. The duration, intensity, complexity and quality of execution of the physical training can be measured. No studies currently exist on exergames and their effects on the cognitive skills of children with ADHD. However, initial investigations with children and adolescents have yielded promising results and therefore seem to demonstrate the usefulness of exergames as an intervention for promoting health-related outcomes [58] and the executive functions [59–62].

### Study aims and hypotheses

The aim of this study is to evaluate the influence of an exergame intervention, which is characterised by both high physical and high cognitive demands, on the executive functions of children with ADHD. We expect that exergaming will have a positive impact on the executive functions, the sport motor performance, the symptoms of ADHD and on quality of life.

As the primary outcome, we hypothesize that exergaming will positively affect executive function performance compared to controls. More specifically, we expect that the demanding exergame activity that will be carried out over a period of 8 weeks will have a beneficial effect on the key areas—inhibition, working memory and cognitive flexibility—and that we will be able to detect a significant effect (significance level α = .05) with small to moderate effect sizes.

As secondary outcomes, we hypothesize that sport motor performance will be significantly improved by the planned intervention (physically strenuous exergaming). As a further secondary outcome, the influence of the intervention on the symptoms of ADHD will be examined. Based on studies into physical training programmes for children and adolescents with ADHD [42], we hypothesize the intervention to have a positive effect. In addition, quality of life is to be measured as a further secondary outcome serving as an indication of a broad transfer. We expect exergaming to have a positive influence on quality of life. The expectations of registering positive developments with respect to the secondary outcomes are generally somewhat more conservative. Based on the available empirical evidence, we therefore expect to obtain small effect sizes.

## Methods/Design

### Study design

The present study will be carried out using a randomised study design with a waiting-list control group in Bern, Switzerland. Each participant in the study will take part in the study for a total of nine weeks, being assigned either to the exergame intervention or to the passive waiting-list control group.

### Participants

In total, 66 children with ADHD between the ages of 8 and 12 years should be examined. Any child that has been diagnosed with the ADHD is eligible to take part in the study. Beyond this, the participants and legal guardians must examine the information relating to the study and give their informed consent to take part in the study. As the well-being of the participants is the main priority, people whose mental or physical integrity might be threatened by the study must not take part in it. For safety reasons, people suffering from a neurological disorder, Tourette syndrome or an epileptic disorder will not be allowed to take part. To recruit the study participants, we will collaborate with elpos Schweiz (an association for parents and caregivers of children and adults with ADHD). The sample size of 66 participants was calculated using G*Power [63] for a general linear model with repeated measures (two comparison groups; three measurements; “within-between interaction”; correlation between measurements = .8), with a power of .80 [64] and a small effect size.

### Intervention

The planned exergame intervention for the experimental group will be carried out using the XBOX Kinect. This device is able to project the player and his or her movements on the screen by means of a camera. It therefore offers the possibility of immersing oneself directly in all sorts of different virtual worlds. The level of physical activity can be increased playfully, and a continuous adjustment to the corresponding level of performance allows the executive functions to be trained. A physically demanding exergame with a strong cognitive component is “Shape UP” (Ubisoft, Montreal, Canada). It is available as a commercial game and resorts to various different elements of endurance, strength and mobility, as well as coordination, working memory, inhibition, attention, speed of action and action planning. In an initial pilot study involving school children, acute effects on cognitive flexibility were noted [62]. In order to achieve the highest possible performance, the player must train both physically and cognitively. The game is designed to be adaptive, automatically adjusting to the player’s level of proficiency. Within the game, there are several different “workouts”, which subjects are supposed to carry out three times a week for at least 30 min, over a period of 8 weeks. The first exergaming session will be conducted under the supervision of the researcher, and at the same time, the intervention will be explained to the children. The parents will be asked to assist and support the children in carrying it out. They too will be given an introduction and brief instructions as to how the game is played. The children and their parents will be informed that they are welcome to contact the study coordinator at any time if they need information or assistance.

### Outcome measures and time-points of assessment

**Fig. 1:**
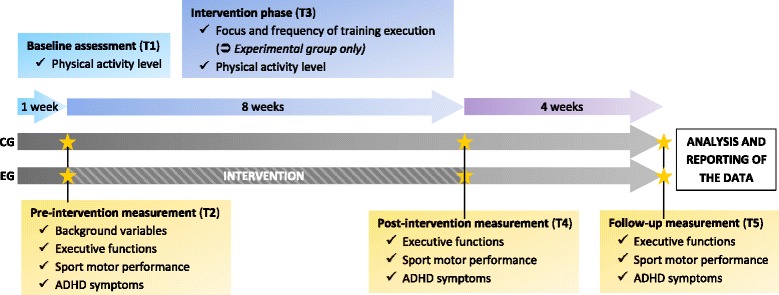
Outcome variables and time points of assessments. CG = Control Group; EG = Experimental Group

The study is divided up into five phases (T1-T5). These are discussed in chronological order below (see Fig. 1):

- Baseline (T1): At the beginning of the study, the participants will be fitted with an accelerometer (Actigraph GT9X), which they are expected to wear during the entire study, to monitor their *physical activity level*. By measuring the acceleration in three-dimensional space, it is for example possible to calculate step counts and MET levels. Participants will be asked to wear the devices at night too, if possible, to obtain data on sleep duration.
- Pre-intervention measurement (T2): The executive functions, sport motor performance, ADHD symptoms, quality of life and background variables will be measured 7 days after handing over the accelerometer. The measures used to determine the *executive functions:* working memory, cognitive flexibility and inhibition, are based on three standardised tests. They are specifically designed for the age group in question and have proven their age adequacy and sensitivity to changes in executive function performance in previous studies [e.g. 51, 65]: working memory performance will be measured using the colour span backwards [66], in which the number of correct answers will be rated. Cognitive flexibility and inhibition will be measured using the adapted Flanker Test [65, 67, 68], again by computer-assisted means. In this, the scores for accuracy and reaction time will be determined. In addition, an adapted version of the Simon Task [69] will be used. The tests have already been successfully used in several studies [51, 65, 70] and proved to be sensitive enough to detect both acute and chronic effects in children between the ages of 8 and 12 years. The cognitive test battery will last approx. 20 min overall. *Sport motor performance* will be measured using the German Motor Test Battery 6–18, which includes eight test items measuring endurance and coordination [71]. Beyond this, the German version of the Physical Activity Enjoyment Scale will be used to measure the subjective pleasure in physical activity [72]. The Shuttle-run Test [73] will be used to measure physical fitness. The *ADHD symptoms* will be ascertained using the German version [74] of the Conners-3 Scales on Attention and Behaviour [75]. These will be completed by parents and teachers. *Quality of life* will be measured using the Inventory of Quality of Life in Children and Adolescents [76]. This questionnaire will be completed by parents and teachers. The *background variables* will be determined using questionnaires and will include the following: age, sex, height, weight, socioeconomic status [77], exercise behaviour [78], and pubertal development [79], completed by the parents.
- Intervention phase (T3): The children in the experimental group will train three times a week for approx. 30 min over a period of 8 weeks. The frequency of training and the accuracy will be recorded by the XBOX Kinect and using a Training Diary. In addition, the exercise activity will be recorded by an accelerometer (over a period of 8 weeks) and the heart rate will be recorded by a cardio belt (only during intervention sessions).
- Post-intervention measurement (T4) and follow-up (T5)*:* At these two times (immediately after the conclusion of the study, and again four weeks later) the tests and questionnaires on the executive functions, quality of life, sport motor performance, attention and behaviour used at T2 will be performed once again.

### Randomisation and blinding

Participants will be randomly assigned to either the intervention or the control group. The randomisation will be conducted in accordance with the regulations of the ethics committee. The teachers will be blinded with respect to treatment allocation, to ensure the data is objective. It will not be possible to blind the researchers and parents since they have to provide guidance on the intervention. Assessments will be performed by two examiners, but as most of the assessments used are objective, the risk of the researcher imposing a bias is small.

### Adverse events

No serious adverse events are expected as a consequence of the treatment. Slight muscle and joint ache might occur and subjects’ eyes may tire. Health and safety precautions that apply to the XBOX Kinect and the exergame must be strictly observed.

### Data analysis

The data collected (background variables, primary and secondary outcomes) will be tested as to their statistical properties and analysed accordingly. Normally distributed background and control variables will be compared between the groups (experimental vs. control group) using the two-sample *t*-test, and non-normally-distributed variables will be tested using a Wilcoxon rank-sum test. A chi-squared test will be used for the categorical variables. Significant differences might be considered as covariates for further analyses.

The diary entries as well as the execution data obtained from the XBOX and the heart rate during training sessions will be used to provide information about the focus and the frequency of the training as well as about the level of physical exertion during exergaming. These scores will be presented as descriptive summaries. The frequency of exercises (including step count, MET level) during the intervention period will then be compared between the groups and analysed using an independent *t*-test.

In order to test the hypotheses, the primary and secondary outcome variables will be analysed using repeated measures ANOVAs, if applicable. To determine whether a repeated measures ANOVA is applicable, the pre-intervention scores will be tested using an independent *t*-test to determine any differences between groups. Provided no significant differences are found, the repeated measures ANOVAs can be performed. If, on the other hand, significant differences are found, an ANCOVA should be carried out on the baseline data to check the differences. In this case, the pre-intervention scores will serve as covariates, and the post-intervention scores will be introduced as dependent variables.

The significance level will be set at *p* < .05 for all analyses. The results will be reported in accordance with the CONSORT guidelines for clinical trials [80].

### Research ethics

The submitted research project has been approved by the ethics committee of the canton of Bern, Switzerland (KEK-NR. 393/15). In addition, the research project has been registered with the Swiss National Clinical Trial Port and the German Register of Clinical Trials (DRKS00010171). After verifying that they are eligible for the study, the participants and their parents will receive adequate verbal information about the study. They will be given enough time to consider their participation and the informed consent information will be sent to them. After at least another seven days, the researcher will visit the families and they will go through the written consent form together. Children above the age of 11 years will be appropriately informed in writing in addition to the oral information. Participation in the study can be terminated at any time without stating the reasons and without detriment to the participant.

## Discussion

The existing empirical evidence indicates that physical activity can have a positive effect on cognitive performance, physical activity and the symptoms of ADHD [31, 42]. In addition, exergame interventions have shown that they are able to increase physical activity and motivation [53]. Particularly in children with ADHD, no scientific findings are currently available from studies that have examined the effects of exergaming.

Various different studies have been able to demonstrate that both physical and cognitive training programmes can have a positive effect on the executive functions [e.g. 27, 28, 44, 45]. Because novelty and diversity are frequently ignored in cognitive trainings, beneficial effects are limited. Therefore, a hybrid between a physical and cognitive training, presented in a child-appropriate form might be a promising approach to foster executive function performance [52]. This is particularly interesting in the light of the fact that children and adolescents with ADHD spend about twice as much time playing video games [81], which could be due to the fact that they prefer the immediate rewards associated with video game play to the delayed rewards associated, for example, with traditional physical activity [82]. A direct form of reward occurs during and immediately after exergaming via the feedback from the computer and the number of points scored. If the effects are found to be positive, it would be desirable to replace some of the time already spent playing video games by exergaming. Thus, the current research project has direct potential applications: if exergaming is able to promote positive effects, it will become useable as an instrument that is already widely available.

From the point of view of society as a whole, fewer and fewer people are engaging in sports and more and more are failing to meet the recommended daily time spent in physical activity [83]. Physical activity is decreasing and many activities are done seated [84]. At the same time, the amount of time spent in front of screens is becoming longer and longer [81], leading to a link between media use, body fatness and physical activity in children and adolescents [85] and reflecting the particular vulnerability of this study population. In addition, particularly children with ADHD have problems taking part in traditional sports programmes and not dropping out of these [86], which can have negative consequences in many areas, because physical inactivity in children and adults has many effects that are detrimental to health and can be seen as a major health factor [87]. New methods are therefore needed to place the focus on health promotion through sports, and in particularly to encourage children and adolescents who are overtaxed by traditional programmes.

A few limitations need to be mentioned, which might affect the results of the study. First of all, a waiting-list control group is planned, ensuring an economic procedure in view of the low level of available empirical evidence. Should the results be favourable, an exergame intervention would have to be compared with a cognitive training and with a regular physical training programme. The planned exergame intervention is a physical training programme with a high cognitive component. The product in question is commercially available so that the entertainment factor is large and the graphics are appealing. Unfortunately, the exergame was not produced and adapted for the specific use in the current study. As a consequence, the cognitive engagement is greater in some of the game’s levels and lower in others. The cognitive engagement of a cognitive training and an optimal challenge point might not be reached all the time.

One of the strengths of the study is its high ecological validity. Children and adolescents with ADHD often play video games. It would therefore seem to be easy to implement active video games at home, and this is being investigated by an increasing number of studies [88]. Furthermore, the study will be randomised and use a large number of control variables, which will allow the sports activity as well as the physical activity of the children to be observed. We therefore expect to also obtain valuable insights beyond the intervention itself.

### Trial status

Recruitment for the trial started in March 2016 and is estimated to be completed by June 2017.

## Abbreviations

ADHD: Attention deficit hyperactivity disorder
ANCOVA: Analysis of covariance
ANOVA: Analysis of variance
CONSORT: Consolidated standards of reporting trials
MET: Metabolic equivalent of task
